# Insulin-like growth factors and related proteins in plasma and cerebrospinal fluids of HIV-positive individuals

**DOI:** 10.1186/s12974-015-0288-6

**Published:** 2015-04-15

**Authors:** Hyeon-Sook Suh, Yungtai Lo, Namjong Choi, Scott Letendre, Sunhee C Lee

**Affiliations:** Department of Pathology, Albert Einstein College of Medicine, Bronx, NY 10461 USA; Department of Epidemiology and Population Health, Albert Einstein College of Medicine, Bronx, NY 10461 USA; Department of Neurology, University of California San Diego, 9500 Gilman Dr, La Jolla, CA 92093 USA

**Keywords:** Biomarker, Growth factor, Cytokines, Neurocognitive impairment, Charter

## Abstract

**Background:**

Clinically significant dysregulation of the insulin-like growth factor (IGF) family proteins occurs in HIV-infected individuals, but the details including whether the deficiencies in IGFs contribute to CNS dysfunction are unknown.

**Methods:**

We measured the levels of IGF1, IGF2, IGFBP1, IGFBP2, and IGF2 receptor (IGF2R) in matching plasma and cerebrospinal fluid (CSF) samples of 107 HIV+ individuals from CNS HIV Antiretroviral Therapy Effects Research (CHARTER) and analyzed their associations with demographic and disease characteristics, as well as levels of several soluble inflammatory mediators (TNFα, IL-6, IL-10, IL-17, IP-10, MCP-1, and progranulin). We also determined whether IGF1 or IGF2 deficiency is associated with HIV-associated neurocognitive disorder (HAND) and whether the levels of soluble IGF2R (an IGF scavenging receptor, which we also have found to be a cofactor for HIV infection *in vitro*) correlate with HIV viral load (VL).

**Results:**

There was a positive correlation between the levels of IGF-binding proteins (IGFBPs) and those of inflammatory mediators: between plasma IGFBP1 and IL-17 (β coefficient 0.28, *P* = 0.009), plasma IGFBP2 and IL-6 (β coefficient 0.209, *P* = 0.021), CSF IGFBP1 and TNFα (β coefficient 0.394, *P* < 0.001), and CSF IGFBP2 and TNF-α (β coefficient 0.14, *P* < 0.001). As IGFBPs limit IGF availability, these results suggest that inflammation is a significant factor that modulates IGF protein expression/availability in the setting of HIV infection. However, there was no significant association between HAND and the reduced levels of plasma IGF1, IGF2, or CSF IGF1, suggesting a limited power of our study. Interestingly, plasma IGF1 was significantly reduced in subjects on non-nucleoside reverse transcriptase inhibitor-based antiretroviral therapy (ART) compared to protease inhibitor-based therapy (174.1 ± 59.8 *vs*. 202.8 ± 47.3 ng/ml, *P* = 0.008), suggesting a scenario in which ART regimen-related toxicity can contribute to HAND. Plasma IGF2R levels were positively correlated with plasma VL (β coefficient 0.37, *P* = 0.021) and inversely correlated with current CD4+ T cell counts (β coefficient −0.04, *P* = 0.021), supporting our previous findings *in vitro*.

**Conclusions:**

Together, these results strongly implicate (1) an inverse relationship between inflammation and IGF growth factor availability and the contribution of IGF deficiencies to HAND and (2) the role of IGF2R in HIV infection and as a surrogate biomarker for HIV VL.

**Electronic supplementary material:**

The online version of this article (doi:10.1186/s12974-015-0288-6) contains supplementary material, which is available to authorized users.

## Background

The insulin-like growth factor (IGF) system consists of growth peptides (IGF1 and IGF2), their receptors (IGF1R and IGF2R), and circulating binding proteins (IGFBPs 1-6) [1-3]. Multiple factors influence IGF levels in biological fluids including nutrition, endocrine status, liver disease, chronic inflammatory diseases, diabetes mellitus, and ethnicity (African American < Asian < Caucasian) [4]. Plasma levels of IGFs are much higher (10 to 100 fold) than required for biological action, as the majority are bound to IGF-binding proteins (IGFBPs) [5,6]. Most circulating IGF1 is produced by the liver, which is under the regulation of pituitary growth hormone (GH). Extrahepatic (non-GH-dependent) IGF1 synthesis also occurs [3]. IGF2 is also produced from hepatic and extrahepatic sources but its production is not tightly regulated by GH [7]. IGF1 and IGF2 are also synthesized in the CNS and surrounding cells [8-10] and have neurotrophic effects [11-14]. Both IGF1 and IGF2 signal through IGF1R, whereas IGF2R is a non-signaling receptor involved in the removal of excess IGF2 during development [15,16]. IGFBPs regulate the biological activity of IGFs by controlling IGF efflux from the vascular space, regulating IGF clearance, and modulating interaction of IGFs with their receptors [17,18]. The liver is a major site of synthesis for IGFBP1. IGFBP1 has been proposed as a dynamic regulator of IGF bioactivity *in vivo*. IGFBP1 may be partly responsible for the decrease in lean body mass observed in catabolic/inflammatory conditions associated with low IGF1. Proinflammatory cytokines can directly enhance IGFBP-1 synthesis [19,20]. IGFBP2 also modulates IGF1 activity [21]. In the brain, IGFBPs are produced by neurons, glial cells, choroid plexus epithelia, and cerebral blood vessels [3]. IGFBP2 is the most abundant IGFBP in the CNS/cerebrospinal fluid (CSF) [18].

There is evidence that reduced IGF1 levels correlate with impaired cognitive function in adults [22,23]. Alzheimer’s patients show increased serum TNFα and decreased IGF1 levels, with the two showing a negative correlation [22]. In a study of motor neuron disease, CSF (but not serum) levels of IGF1 were diminished [23]. Uptake of circulating IGF1 into the CSF can be substantial and circulating IGF1 could aid brain function [24,25]. This may explain why circulating IGF1 levels correlate with deterioration of cognitive functioning in elderly patients [26].

IGFs and related factors have been studied in the context of HIV infection and associated diseases, as they may play a role in the immune response to HIV and influence the rate of HIV disease progression [27,28]. HIV/AIDS is associated with reduced IGF1 and IGF2 levels [7,28]. Dysregulation of IGF1 also contributes to metabolic syndromes and lipodystrophy in these individuals [7,29,30]. IGFBP1 and IGFBP2 may also function as proinflammatory factors correlating with HIV disease activity [31-33]. The effect of antiretroviral therapy (ART) on IGF proteins can also be substantial [34,35]. Specifically, certain types of ART have been linked to lipodystrophy [36,37], vitamin D deficiency, and reduced serum IGF1 levels [38,39].

Little or no information is available on the role of IGFs and related proteins in neuroAIDS. HIV-associated neurocognitive disorder (HAND) affects approximately 50% of HIV+ individuals through several different mechanisms [40-42]. Intact virus and viral components as well as host inflammatory mediators and ART have all been implicated. There is a pressing need to identify clinically useful biomarkers for HAND. Studies by this and other laboratories suggest that depletion of neuronal growth factors might contribute to the development of HAND. Specifically, we have shown that the essential CNS growth factor progranulin is deficient in the CSF of HIV-undetectable adults with neurocognitive impairment (NCI) but not HIV-detectable adults with HAND [43,44]. Both progranulin and IGF1 are produced by microglia and modulated by inflammatory mechanisms [13,45]. A related protein IGF2 can replace IGF1 for IGF1R signaling and neuroprotection [13]. IGF2R that is normally expressed by neurons alone in the CNS is highly upregulated in microglia in HIV encephalitis and functions as a cofactor for HIV infection [46]. High levels of (soluble) IGF2R are present in blood, urine, and amniotic fluid [47,48], suggesting its potential usefulness as a biomarker.

The purpose of this study was to determine whether IGF1 or IGF2 deficiency exists in HIV+ individuals, especially in the context of neurocognitive dysfunction (HAND) and to examine the relationship between IGFs (IGF1 and IGF2) and IGFBPs (IGFBP1 and IGFBP2) in a well-characterized HIV+ cohort. We hypothesized that reduced levels of IGF1 in plasma or CSF (or both) would be associated with HAND and that chronic inflammation state in HIV infection would contribute to this. In addition, given the novel finding that IGF2R is upregulated in activated microglia and macrophages and functions as an HIV cofactor, we examined the association between IGF2R levels and HIV viral load (VL).

## Methods

### CHARTER subjects

CSF and plasma pairs were collected from 107 adults enrolled in the cross-sectional component of the CNS HIV Antiretroviral Therapy Effects Research (CHARTER) cohort, a six-center, US-based project funded by the National Institutes of Health. The project was approved by the institutional review board (IRB) of each participating institution (Johns Hopkins University, Mt. Sinai School of Medicine, University of California, San Diego, University of Texas Medical Branch-Galveston, University of Washington, and Washington University), as well as Albert Einstein College of Medicine. All participants provided written informed consent. Individuals with abnormal serum glucose, HCV seropositivity, or current substance use (based on urine drug screening) were excluded. Individuals receiving human growth hormone (GH), GH releasing factor, or hematopoietic growth factors such as erythropoietin were also excluded.

### Neuropsychological testing

All participants completed a comprehensive neurocognitive test battery (speed of Information processing, learning and memory, executive functions, language, working memory, motor, self-report questionnaires, psychiatric interviews), covering seven cognitive domains that are commonly affected by HIV. The best available normative standards were used, which correct for effects of age, education, gender, and ethnicity, as appropriate. Test scores were automatically converted to demographically corrected standard scores (T-scores) using available computer programs. To classify presence and severity of neurocognitive impairment, a published objective algorithm that has been shown to yield excellent interrater reliability in previous multisite studies [49] was applied. This algorithm conforms to the Frascati criteria for diagnosing HAND [42], which requires presence of a least mild impairment in at least two domains.

### IGF1 ELISA

IGF1 levels were quantified by enzyme-linked immunosorbent assay (ELISA, ALPCO Diagnostics, Salem, NH, USA: cat # 22-IGFHU-E01, sensitivity 90 pg/ml) using the core service of the Einstein Diabetes Research Center (Biomarker Analytic Research Core). We measured total IGF1 levels, that is, free and IGFBP-associated, following the manufacturer’s instructions. Briefly, ALPCO kits separate IGF1 from IGFBPs by acidification. Re-association of IGF1 with IGFBPs (after restoration of neutral pH) is prevented with an excess of IGF2. IGF2 blocks the access of IGF1 to the IGFBP binding sites without interfering with the IGF1-specific antibodies used in the assay. Samples were assayed in duplicate.

### IGF2 ELISA

IGF2 levels were also quantified by ELISA (ALPCO Diagnostics, NH: cat # 22-IGF-E30, sensitivity 20 pg/ml) using the core service of the Einstein Diabetes Research Center (Biomarker Analytic Research Core, Bronx, NY, USA). Similar to IGF1 ELISA, results do not depend on the binding protein concentration of the sample. There is no cross-reactivity with IGF1. IGF2 was separated from the binding proteins by acidification, and re-association of the freed binding proteins with IGF2 was prevented by excess IGF1. Samples were assayed in duplicate.

### IGF2R ELISA

Soluble IGF2R levels were quantified by ELISA (R&D Systems, Minneapolis, MN, USA: cat #DY 2447, sensitivity approximately 150 pg/ml), following the manufacturer’s instructions. The samples were diluted until the values fell within the linear range of detection (approximately 1:80 for plasma and approximately 1:10 for CSF).

### Other biomarker quantifications

Concentrations of inflammatory mediators were determined in the plasma and CSF samples of all 107 subjects, as previously reported [43]. A multiplex bead array quantified concentrations of IFNγ, IL-1β, IL-4, IL-6, IL-10, IL-13, IL-17, TNFα, CCL2/MCP-1, CXCL10/IP-10, IGFBP1, and IGFBP2 (EMD Millipore, Billerica, MA, USA). Samples were diluted until the final concentrations were within the linear range of detection for the assay. Progranulin was measured by ELISA (DY2420, R&D Systems), as reported [43]. All samples were tested in duplicate and concentrations interpolated from a standard curve constructed by four-parameter fitting of internal standards.

### Data analysis

Inflammatory mediators whose levels were below the detection limit of the assay in >90% of samples were excluded from analysis. These were IL-1β, IL-4, and IL-13 for plasma and IL-1β, IL-4, IL-13, IFNγ, and IL-17 for CSF. Undetectable HIV VL designates HIV RNA levels below 50 copies/ml, the lower limit of quantification (Amplicor, Roche Diagnostics, Indianapolis, IN, USA).

For normalization, plasma IGFBP1, plasma IGF2R, CSF IGF2, and CSF IGFBP2 concentration values were transformed with natural logarithms, and CSF IGF2R values were square-root transformed. Differences in demographic characteristics, AIDS status, current CD4 count, HIV VL, alcohol dependence, albumin, AST, ALT, and IGF1 levels in the plasma and CSF between HIV-infected individuals with and without neurocognitive impairment were examined using *t*-tests or Wilcoxon rank-sum tests for continuous variables and using chi-squared or Fisher’s exact tests for categorical variables. Initial analysis of associations of demographic and disease characteristics with plasma and CSF IGF protein concentrations were examined using *t*-tests or Wilcoxon rank-sum tests for categorical variables and Spearman correlation coefficients for continuous variables. Linear regression models or analysis of covariances (ANCOVA) were used to examine associations of IGF proteins with neurocognitive status adjusted for demographics, HIV VL, and inflammatory mediators. Variables with a *P*-value ≤0.2 in initial analysis were included in multivariate analysis. Because neither log-transformation nor square root transformation normalized CSF IGF1, CSF IGFBP1, or plasma IGFBP2, non-parametric analysis of covariances was used for these three variables. Statistical analyses were performed using SAS (version 9.1, SAS Institute, Cary, NC, USA) software.

## Results

### Demographic and clinical characteristics of HIV+ individuals in the cohort

The demographic and clinical characteristics of HIV+ individuals in the cohort are listed in Table 1. This cohort is identical to that used for our recent progranulin study [43]. Additional factors that are relevant to IGF analysis (such as BMI, liver function tests) are also listed. Individuals with HCV infection are excluded from the study. When the characteristics were stratified by NC impairment, there were no significant differences between individuals with and without NC impairment, except age. Individuals with NC impairment were older than those without NC impairment (45.3 ± 9.3 *vs*. 41.8 ± 7.6, *P* = 0.033) (Table 1).

**Table 1:** **Demographic and clinical characteristics (combined and stratified by neurocognitive impairment)**

**Characteristics**	**All subjects**	**NC impaired**	**NC unimpaired**	
	**(** ***n*** **= 107)**	**(** ***n*** **= 58)**	**(** ***n*** **= 49)**	***P*** **value**
Age, years - mean ± SD	43.7 ± 8.7	45.3 ± 9.3	41.8 ± 7.6	0.033
Gender, male - *N* (%)	93 (86.9%)	52 (89.7%)	42 (85.7%)	0.406
Race				
White - *N* (%)	59 (55.1%)	31 (53.5%)	28 (57.1%)	0.962
Black	34 (31.8%)	18 (31.0%)	16 (32.7%)	
Other	14 (13.1%)	9 (15.5%)	5 (10.2%)	
AIDS - *N* (%)	68 (63.6%)	38 (65.5%)	30 (61.2%)	0.554
ART use - *N* (%)	84 (78.5%)	45 (77.6%)	39 (79.6%)	0.956
Plasma HIV VL undetectable - *N* (%)	53 (49.5%)	29 (50.0%)	24 (50.0%)	0.838
CSF HIV VL undetectable - *N* (%)	79 (73.8%)	44 (75.9%)	36 (73.5%)	0.910
Current CD4+ count (<200 cells/μL) - *N* (%)	22 (20.8%)	9 (15.5%)	13 (26.5%)	0.160
Current CD4+ count - median (IQR)	405 (216 to 582)	435 (260 to 589)	360 (175 to 531)	0.247
Albumin (<5 g/dL)	72 (67.9%)	37 (64.9%)	35 (71.4%)	0.474
Alcohol dependence - *N* (%)	25 (23.2%)	12 (20.7%)	13 (26.5%)	0.514
HGB (mean ± SD)	14.1 ± 1.5	14.1 ± 1.6	14.1 ± 1.4	0.882
AST - median (IQR)	30 (23 to 37)	31 (24 to 39)	30 (22 to 36)	0.673
ALT - median (IQR)	33 (22 to 43)	36 (23 to 43)	30 (22 to 40)	0.298
Body mass index (BMI)				0.290
BMI ≤ 25	45 (42.1%)	24 (41.4%)	21 (42.9%)	
25 < BMI < 30	45 (42.1%)	22 (37.9%)	23 (46.9%)	
BMI ≥ 30	17 (15.8%)	12 (20.7%)	5 (10.2%)	

### Measurement of IGFs and related proteins in plasma and CSF

The raw values for the IGFs and related proteins measured in plasma and CSF are listed in Table 2. As these factors are infrequently measured, especially in CSF, we listed the available references for comparison. The levels measured in our study are in general agreements with those reported. The median plasma IGF1 and IGF2 levels had the known ratio of approximately 1:3. The CSF IGF1 levels were extremely low, representing approximately 1/200 of the plasma level. The CSF IGF2 and IGFBP1 were approximately 1/20 and approximately 1/7 of the plasma levels, respectively, and the CSF IGFBP2 levels were approximately 2.3-fold higher than plasma levels, suggesting that IGF2 and IGFBP2 play more significant roles in the CNS relative to IGF1 and IGFBP1. The median CSF IGF2R level was 6.65 ng/ml and represented approximately 1/20 of the plasma levels. To our knowledge, this is the first reported measurement of IGF2R levels in CSF.

**Table 2:** **IGFs and related protein levels in the plasma and CSF**

**Factors**	**Plasma**		**CSF**		**Ratio (CSF:plasma)**
	**Median (IQR) (ng/ml)**	**References**	**Median (IQR) (ng/ml)**	**References**	
IGF1	195.3 (165.0 to 224.8)	[57,60-63]	1.11 (0.92 to 1.48)	[61,64,65]	1:134
IGF2	693.9 (587.2 to 817.0)	[60,62,64,66]	40.26 (34.6 to 45.4)	[64,67]	1:17
IGFBP1	3.95 (2.53 to 7.01)	[62,63,68]	0.48 (0.35 to 0.77)	[68]	1:7
IGFBP2	33.97 (14.44 to 51.76)	[60,62]	77.03 (59.3 to 101.2)	[64,67]	1:0.4
IGF2R	99.73 (52.3 to 176.3)	[47,48,69,70]	6.65 (3.79 to 10.00)	None	1:17

IQR, interquartile range.

### IGFs and related proteins in subjects with or without NC impairment

We next compared the levels of IGFs and related proteins in subjects with or without NC impairment to determine whether the levels of any of these factors differ in the two groups. The results showed no significant differences in plasma or CSF IGFs in the two groups (Table 3). Although the median CSF IGF1 levels were reduced in the subgroup with NC impairment compared with non-impaired (1.11 *vs*. 1.13), this was far from significant (*P* = 0.821). Similarly, both the plasma IGF1 (186.0 *vs*. 200.1 *P* = 0.127) and IGF2 (686.1 *vs*. 723.3, *P* = 0.520) were reduced in the subgroup with NP impairment but without significance (see the ‘Discussion’ section).

**Table 3:** **Comparison of IGFs and related proteins in plasma and CSF in subjects with or without neurocognitive impairment**

	**Factors**	**All subjects**	**NC impaired**	**NC unimpaired**	***P*** **value**
		**(** ***n*** **= 107)**	**(** ***n*** **= 58)**	**(** ***n*** **= 49)**	
Plasma	IGF1 (mean ± SD, ng/ml)	192.5 ± 55.3	186.0 ± 63.28	200.1 ± 43.7	0.127
	IGF2 (mean ± SD, ng/ml)	703.3 ± 165.2	686.1 ± 170.5	723.3 ± 158.3	0.520
	log IGFBP1 (mean ± SD, ng/ml)^a^	1.43 ± 0.74	1.45 ± 0.85	1.40 ± 0.59	0.797
	IGFBP2 - median (IQR)	33.97	38.59	26.00	0.346
		(14.44 to 51.76)	(16.13 to 47.72)	(6.54 to 54.39)	
	log IGF2R (mean ± SD, ng/ml)^a^	4.51 ± 0.88	4.47 ± 0.97	4.56 ± 0.77	0.716
CSF	IGF1 - median (IQR)	1.11 (0.92 to 1.48)	1.11 (0.88 to 1.48)	1.13 (0.92 to 1.51)	0.821
	log IGF2 (mean ± SD, ng/ml)^a^	3.70 ± 0.17	3.72 ± 0.17	3.68 ± 0.17	0.345
	IGFBP1 - median (IQR)	0.48 (0.35 to 0.77)	0.50 (0.37 to 0.75)	0.47 (0.33 to 1.59)	0.880
	log IGFBP2 (mean ± SD, ng/ml)^a^	4.35 ± 0.37	4.35 ± 0.32	4.36 ± 0.41	0.986
	IGF2R^0.5^ (mean ± SD, ng/ml)^b^	2.56 ± 0.84	2.51 ± 0.87	2.62 ± 0.80	0.498

^a^Plasma IGFBP1, plasma IGF2R, CSF IGF2 and CSF IGFBP2 were transformed with natural logarithms; ^b^CSF IGF2R was square root transformed. IQR, interquartile range.

### Analyses of plasma proteins

#### Multivariate analyses of factors associated with plasma IGFs and related proteins

In order to examine the relationship between plasma IGF protein expression and the demographic and clinical factors as well as inflammation, we initially performed (1) univariate analyses of factors associated with plasma IGFs and related proteins and (2) correlation analyses of IGF proteins and inflammatory mediators as described under the ‘Methods’ section. The results are presented in Additional file 1: Table S1A and S1B, respectively. We additionally performed multivariate analyses for each of the plasma IGF proteins to determine significant associations. The results are shown in Tables 4, 5, 6, 7, and 8. Table 4 shows multivariate analysis for plasma IGF1. Men had higher plasma IGF1 compared to women (β coefficient = 31.81, *P* = 0.039). Older age (β coefficient = −1.31, *P* = 0.033), AIDS (β coefficient = −18.91, *P* = 0.081) and higher AST (β coefficient = −0.36, *P* = 0.026) were associated with lower plasma IGF1. NC impairment was not associated with plasma IGF1. Table 5 shows multivariate analysis for plasma IGF2. Lower IP-10 (β coefficient = −125.0, *P* = 0.017) and alcohol dependence (β coefficient = 71.8, *P* = 0.023) were associated with higher plasma IGF2. NC impairment was not associated with plasma IGF2. Table 6 shows multivariate analysis for plasma IGFBP1. Higher IL-17 was associated with higher plasma IGFBP1 (β coefficient = 0.28, *P* = 0.009). Plasma IGFBP1 was lower in subjects with 25 < BMI < 30 (β coefficient = −0.46, *P* = 0.001) or subjects with BMI ≥ 30 (β coefficient = −0.94, *P* <0.001) compared to subjects with BMI ≤ 25. Table 7 shows non-parametric multivariate analysis of plasma IGFBP2. Plasma IGFBP2 was higher in whites compared to blacks or other race (*P* = 0.027). Higher IL-6 was associated with higher plasma IGFBP2 (*P* = 0.021). NC impairment was not associated with plasma IGFBP2. Multivariate linear regression analysis of factors associated with plasma IGF2R is shown in Table 8. Men had lower plasma IGF2R compared to women (β coefficient = −0.48, *P* = 0.042). Detectable plasma VL (β coefficient = 0.37, *P* = 0.021) and lower CD4 count (β coefficient = −0.04, *P* = 0.021) were associated with higher plasma IGF2R. Higher IL-10 (β coefficient = 0.18, *P* = 0.061) was associated with higher plasma IGF2R, but this result is borderline significance. NC impairment (β coefficient = −0.03, *P* = 0.859) was not associated with plasma IGF2R.

**Table 4:** **Multivariate linear regression analysis of factors associated with plasma IGF1**

**Plasma characteristics**	**β coefficient (S.E.)**	***P*** **value**
Neurocognitive impairment	−8.48 (10.45)	0.418
Gender, male	31.81 (15.2)	0.039
Age	−1.31 (0.61)	0.033
AIDS	−18.91 (10.74)	0.081
AST	−0.36 (0.16)	0.026

β coefficients less than zero indicate an inverse association between the characteristic and plasma IGF1.

**Table 5:** **Multivariate linear regression analysis of factors associated with plasma IGF2**

**Plasma characteristics**	**β coefficient (S.E.)**	***P*** **value**
Neurocognitive impairment	−21.9 (31.0)	0.482
IP-10, per log_10_	−125.0 (51.5)	0.017
Alcohol dependence	71.8 (31.3)	0.023

β coefficients less than zero indicate an inverse association between the characteristic and plasma IGF2.

**Table 6:** **Multivariate linear regression analysis of factors associated with plasma IGFBP1**

**Plasma characteristics**	**β coefficient (S.E.)**	***P*** **value**
Neurocognitive impairment	0.14 (0.13)	0.261
IL-17, per log_10_	0.28 (0.11)	0.009
25 < BMI < 30 (reference: BMI ≤25)	−0.46 (0.14)	0.001
BMI ≥30 (reference: BMI ≤ 25)	−0.94 (0.18)	<0.001

β coefficients less than zero indicate an inverse association between the characteristic and plasma IGFBP1.

**Table 7:** **Non-parametric analysis of covariance of factors associated with plasma IGFBP2**

**Plasma characteristics**		**Median (IQR)**	***P*** **value**
Neurocognitive impairment			0.624
	Yes	38.59 (16.53 to 47.72)	
	No	26.0 (6.54 to 54.39)	
Race			0.027
	Black	24.57 (3.90 to 45.44)	
	Other	15.08 (6.54 to 44.26)	
	White	40.84 (22.09 to 54.39)	
IL-6		0.209^a^	0.021

^a^Spearman correlation coefficient between IL-6 and plasma IGFBP2. IQR, interquartile range.

**Table 8:** **Multivariate linear regression analysis of factors associated with plasma IGF2R**

**Plasma characteristics**	**β coefficient (S.E.)**	***P*** **value**
Neurocognitive impairment	−0.03 (0.16)	0.859
Gender, male	−0.48 (0.23)	0.042
Current CD4+ count, per 50 copies	−0.04 (0.02)	0.021
Detectable HIV VL	0.37 (0.16)	0.021
IL-10, per 10 units	0.18 (0.10)	0.061

β coefficients less than zero indicate an inverse association between the characteristic and plasma IGF2R.

### Analyses of CSF proteins

#### Multivariate analyses of factors associated with CSF IGFs and related proteins

Univariate analyses of factors associated with CSF IGFs and related proteins, as well as correlation analyses of CSF IGFs, related proteins and inflammatory mediators are presented in Additional file 1: Table S2A and S2B, respectively. Multivariate analyses of CSF IGF proteins are shows in Table 9. Table 9 shows multivariate analysis for CSF IGF1. Whites had higher CSF IGF1 compared to blacks or other race (*P* = 0.002). No association between CSF IGF1 and NC impairment was found. Table 10 shows multivariate analysis for CSF IGF2. Higher IP-10 (β coefficient = 0.11, *P* < 0.001) and ART use (β coefficient = 0.14, *P* < 0.001) were associated with higher CSF IGF2. Blacks (β coefficient = −0.12, *P* < 0.001) or other race (β coefficient = −0.13, *P* = 0.004) were associated with lower CSF IGF2. Alcohol dependence was also associated with lower CSF IGF2 (β coefficient = −0.07, *P* = 0.029). There was no association between CSF IGF2 and NC impairment. Table 11 shows non-parametric analysis of covariance of factors associated with CSF IGFBP1. CSF IGFBP1 was higher in subjects with AIDS (*P* = 0.016) and alcohol dependence (*P* = 0.037). Higher CSF TNFα was associated with higher CSF IGFBP1 (*P* < 0.001). Higher BMI was associated with lower CSF IGFBP1 (*P* < 0.001). No association was found with NC impairment. Table 12 shows multivariate analysis for CSF IGFBP2. Higher CSF TNFα was associated with higher CSF IGFBP2 (β coefficient = 0.14, *P* < 0.001). No association was found with other factors. Table 13 shows multivariate analysis for CSF soluble IGF2R. Older age was associated with higher CSF IGF2R (β coefficient = 0.03, *P* = 0.002). Alcohol dependence was associated with lower CSF IGF2R (β coefficient = −0.35, *P* = 0.049). Blacks had lower CSF IGF2R compared to whites (β coefficient = −0.31, *P* = 0.068) while other race had higher CSF IGF2R compared to white (β coefficient = 0.49, *P* = 0.035). There was no significant association between CSF IGF2R and NC impairment.

**Table 9:** **Non-parametric analysis of covariance of factors associated with CSF IGF1**

**CSF characteristics**	**Median (IQR)**	***P*** **value**
Neurocognitive impairment		
Yes	1.11 (0.88 to 1.48)	0.557
No	1.18 (0.92 to 1.48)	
Race		
Black	0.92 (0.73 to 1.29)	0.002
Other	1.09 (0.95 to 1.48)	
White	1.22 (0.99 to 1.71)	
Alcohol dependence		
No	1.07 (0.88 to 1.48)	0.042
Yes	1.29 (1.03 to 1.82)	
Age	0.16^a^	0.075

^a^Spearman correlation coefficient between age and CSF IGF1. IQR, interquartile range.

**Table 10:** **Multivariate linear regression analysis of factors associated with CSF IGF2**

**CSF characteristics**	**β coefficient (S.E.)**	***P*** **value**
Neurocognitive impairment	0.02 (0.03)	0.429
Black race (reference = White)	−0.12 (0.03)	<0.001
Other race (reference = White)	−0.13 (0.04)	0.004
ART use	0.14 (0.04)	<0.001
IP-10, per log_10_	0.11 (0.02)	<0.001
Alcohol dependence	−0.07 (0.03)	0.029

β coefficients less than zero indicate an inverse association between the characteristic and CSF IGF2.

**Table 11:** **Non-parametric analysis of covariance of factors associated with CSF IGFBP1**

**CSF characteristics**		**Median (IQR)**	***P*** **value**
Neurocognitive impairment			0.613
	Yes	0.49 (0.37 to 0.75)	
	No	0.47 (0.33 to 1.62)	
AIDS			0.016
	Yes	0.49 (0.36 to 1.17)	
	No	0.44 (0.34 to 0.65)	
Body mass index (BMI)			<0.001
	BMI ≤ 25	0.60 (0.46 to 1.53)	
	25 < BMI < 30	0.41 (0.33 to 0.66)	
	BMI ≥ 30	0.37 (0.23 to 0.40)	
Alcohol dependence			0.037
	Yes	0.64 (0.40 to 1.62)	
	No	0.43 (0.34 to 0.72)	
Albumin			0.061
	3 or 4	0.48 (0.37 to 1.50)	
	5	0.44 (0.30 to 0.62)	
TNF-α		0.394^a^	<0.001
Current CD4 Count		−0.044^b^	0.067

^a^Spearman correlation coefficient between TNF-α and CSF IGFBP1; ^b^Spearman correlation coefficient between current CD4 count and CSF IGFBP1. IQR, interquartile range.

**Table 12:** **Multivariate linear regression analysis of factors associated with CSF IGFBP2**

**CSF characteristics**	**β coefficient (S.E.)**	***P*** **value**
Neurocognitive impairment	0.01 (0.07)	0.861
TNF-α, per log_10_	0.14 (0.03)	<0.001

β coefficients less than zero indicate an inverse association between the characteristic and CSF IGFBP2.

**Table 13:** **Multivariate linear regression analysis of factors associated with CSF IGF2R**

**CSF characteristics**	**β coefficient (S.E.)**	***P*** **value**
Neurocognitive impairment	−0.24 (0.15)	0.121
Age, per year	0.03 (0.01)	0.002
Black race (reference = White)	−0.31 (0.17)	0.068
Other race (reference = White)	0.49 (0.23)	0.035
Alcohol dependence	−0.35 (0.18)	0.049

β coefficients less than zero indicate an inverse association between the characteristic and CSF IGF2R.

#### Multivariate analyses of IGFs stratified by ART regimen

We analyzed whether IGF protein levels differ based on the ART regimen. Of the 86 subjects with ART, 36 received non-nucleotide reverse transcriptase inhibitors (NNRTI)-based regimen and 40 received protease inhibitors (PI)-based regimen, and 8 received combined regimen. Univariate analyses of IGFs stratified by ART regimen are presented in Additional file 1: Table S3, and multivariate analyses are shown in Table 14. The results show that mean plasma IGF1 levels were lower in subjects on NNRTI-based regimen compared to those on PI-based regimen (174.1 ± 59.8 *vs*. 202.8 ± 47.3), and this difference was highly significant on multivariate analysis (*P* = 0.008). Other plasma IGF proteins did not differ significantly in the two subgroups. There were no significant differences in CSF IGF proteins between subjects on NNRTI-based regimen and those on PI-based regimen.

**Table 14:** **Multivariate linear regression analysis of factors associated with plasma IGF1 in 76 subjects on ART**

**Plasma characteristics**	**β coefficient (S.E.)**	***P*** **value**
Neurocognitive impairment	−6.25 (12.37)	0.615
AIDS	−29.01 (15.89)	0.072
PI^a^	34.33 (12.61)	0.008
AST	−0.97 (0.46)	0.036

β coefficients less than zero indicate an inverse association between the characteristic and plasma IGF1. ^a^37 on NNTRI-based and 40 on PI-based regimen (see text).

## Discussion

Our analyses of IGF proteins included association with demographic and clinical parameters which confirmed many of the previously known associations, such as with age, gender, race, BMI, and liver function. As these factors have rarely been examined in the CSF, our study also provides useful information in comparison to their plasma levels. For example, while plasma IGF1 levels decreased with aging (as reported [26]), CSF IGF2R (as well as IGF1 and IGF2 levels) tended to *increase* with aging. Black race was associated with lower plasma levels of IGF family proteins (as reported [50,51]), and their levels (IGF1, IGF2, and IGF2R) measured in the CSF were also lower in Blacks. Lower IGFBP1 levels in both plasma and CSF were strongly (*P* < 0.001) associated with high BMI [52]. Alternations of many CSF IGF family proteins were also associated with alcohol dependence. While we cannot explain exact mechanisms for these associations, our data suggest that IGF proteins play dynamic and complex roles under physiologic and pathologic conditions and form the basis for future studies.

There are well-known associations between certain IGF family proteins, suggesting that their productions are coordinately regulated. Our study confirms some of these associations and provides additional information on their relationships (see the ‘Results’ section including Additional file 1). For example, in plasma, IGF1 and IGF2 were closely correlated and IGFBP1 and IGFBP2 were closely correlated (Additional file 1: Table S1B). Inflammatory mediators showed positive correlations with IGFBP1/IGFBP2 and negative correlations with IGF1/IGF2, indicating that inflammation suppresses IGF1/IGF2 production (Additional file 1: Table S1B). The inflammatory mediators that showed correlations included IL-6, which is an established biomarker for systemic inflammation in HIV+ individuals [53], as well as the newly defined proinflammatory cytokine IL-17. These findings together suggest that inflammation is a highly significant underlying mechanism that regulates plasma IGF protein levels. IGF2R, which has not been examined in the context of HIV until now, showed a correlation with IGFBP1 and IGFBP2, as well as all of the inflammatory mediators examined (IL-17, IFNγ, IL-10, TNF-α, IL-6, and MCP-1) except IP-10 on univariate analyses (Additional file 1: Table S1B). These results suggest that plasma IGF2R is induced by inflammatory mediators and is a potential biomarker for systemic inflammation in HIV+ individuals.

In our study, we also find that ART use was associated with alternations of plasma and CSF IGF protein levels. Specifically, higher plasma IGF2, CSF IGF2, and CSF IGF2R levels were associated with ART use on univariate analysis (Additional file 1: Table S1A and Table 4), possibly reflecting virologic and immunologic improvements associated with ART. Another important change in the IGF axis relates to the types of ART. For example, in our cohort, lower IGF1 levels were strongly associated with non-nucleotide reverse transcriptase inhibitors (NNRTI)-based regimen compared to protease inhibitors (PI)-based regimen (*P* = 0.008). This is consistent with the notion that efavirenz-induced vitamin D deficiency caused circulating IGF-1 deficiency in our cohort, as (1) efavirenz has been reported to induce vitamin D deficiency [39], (2) vitamin D is a known modulator of circulating IGF1 [38], and (3) efavirenz is the main NNRTI used by CHARTER subjects. These results add significantly to the notion that toxicity related to ART can contribute to neurocognitive impairment, in part, through growth factor depletion.

Analyses of CSF revealed that IGF2R levels correlated with IGF2 but not with other proteins or with inflammatory mediators (Additional file 1: Table S2B). These results may reflect that the source of CSF IGF2R in this cohort is mostly neuronal with little or no contributions from microglia or macrophages. This is consistent with our previous study which showed that *de novo* IGF2R induction in microglia is found in HIV encephalitis but not in most HIV+ (but HIVE-) brains [46]. In the CSF, IGF2 appears to play a more significant role than IGF1. First, the IGF2: IGF1 ratios were considerably higher in the CSF (approximately 40:1) than plasma (approximately 3:1), indicating intrathecal production. Second, CSF IGF2 showed correlations with most other CSF factors including IGF2R, IGF1, IGFBP1, IGFBP2, IL-10, IP-10, and TNFα (Additional file 1: Table S2B). Furthermore, CSF IGFBP1 correlated with CSF IGFBP2, and both IGFBPs correlated with inflammatory mediators. The correlations were most significant with IGFBP2, whose CSF levels were higher than those in plasma, again indicating intrathecal production. The emerging picture is then IGF2 and IGFBP2 are the major IGF family proteins in the CSF/CNS. The source of IGF2 in the CSF compartment includes the choroid plexus and the meninges [14]. We found *in vitro* that while both IGFs were equally neuroprotective, IGF2 was *up*regulated and IGF1 was *down*regulated by proinflammatory mediators in cultured microglia [13], suggesting that the two IGFs may not (necessarily) be co-regulated in the CNS. The potential source of IGF1 in the brain and CSF compartment is also multiple including monocyte-lineage cells and other mesenchymal cells [13]. However, because intrathecal IGF1 production is minimal compared to systemic circulation, the most important source of CSF IGF1 might be circulating IGF1 [18]. Furthermore, as IGF2R is a scavenging receptor for both IGFs, upregulated IGF2R expression in CSF/CNS (in HIV encephalitis, for instance [46]) could function as a significant modulator of IGF protein expression. For instance, soluble IGF2R shed from cells (such as activated microglia and macrophages) can capture IGF2 and IGF1, effectively blocking their binding to cognate signaling receptor IGF1R. In this regard, the soluble IGF2R we detect in plasma/CSF should be considered a kind of IGF1R antagonist.

Our study confirms some of the reported IGF axis alterations associated with HIV infection and also adds new data. The association of low plasma IGF1 (or IGF2) and high IGFBPs with HIV progression (AIDS, low CD4 T cell counts) have been well established [28,31-33,54-56]. We also show for the first time that increased plasma IGF2R is associated with detectable plasma VL and low CD4+ T cell counts. As *de novo* IGF2R expression occurs in human microglia in HIV encephalitis (a model of tissue macrophages HIV infection) and IGF2R is a cellular cofactor for HIV replication [46], our biomarker data supports that IGF2R (likely induced by inflammatory mediators) boosts HIV replication (hence detectable VL) and induces CD4+ T cell depletion. Together, these results show a novel finding that IGF2R is an important new player in the IGF axis that connects inflammation, HIV replication, and growth factor depletion.

We initially hypothesized that lower CSF IGF1 might be associated with NC impairment in HIV+ individuals but our hypothesis has not been borne out by our analyses. There are several possible reasons for this. First, the CSF IGF1 levels are very low (approximately 1/100 to 1/200 of plasma levels) and therefore proving our hypothesis may require a much larger cohort. Second, the IGF axis has been shown to exhibit great inter-individual heterogeneity. For example, longitudinal analysis of 1,422 HIV+ women showed only a trend for association between low IGF1 levels and HIV disease progression [57]. In our analysis of 107 HIV+ subjects, plasma IGF1 and IGF2 levels as well as CSF IGF1 levels were lower in NC impaired (*n* = 58) compared to those in NC unimpaired (*n* = 49) subjects, though they were not statistically significant. Of the three, plasma IGF1 showed the largest difference between the two groups (186.5 ± 63.28 *vs*. 200.1 ± 43.7, *P* = 0.127), possibly suggesting that a much larger cohort may have shown a significant association between low plasma IGF1 and NC impairment. Furthermore, a combined growth factor deficiency (rather than a single deficiency) may contribute to significant functional outcome. In this regard, positive correlation between CSF IGF1 and CSF progranulin in our cohort (*r* = 0.26, *P* = 0.008) (Additional file 1: Table S2B) suggests a common mechanism of growth factor regulation in HIV+ individuals and lend further support to this hypothesis. Given the number of studies hinting at the role of CNS growth factor deficiencies in HAND [43,58,59], further studies are warranted to examine their potential roles in the pathogenesis and this complex CNS disorder.

## Additional file

Additional file 1:
**Univariate analyses of factors associated with plasma and CSF insulin-like growth factors and related proteins.**
