# Supplementary material for: Insulin-like growth factors and related proteins in plasma and cerebrospinal fluids of HIV-positive individuals
Source: J Neuroinflammation. 2015 Apr 15;12:72. doi: 10.1186/s12974-015-0288-6 (PMC4407382; doi:10.1186/s12974-015-0288-6)
Supplement: Additional file 1: — Univariate analyses of factors associated with plasma and CSF insulin-like growth factors and related proteins. [file 12974_2015_288_MOESM1_ESM.docx]

**SUPPLEMENTARY DATA:**

**UNIVARIATE ANALYSES OF FACTORS ASSOCIATED WITH PLASMA AND CSF INSULIN-LIKE GROWH FACTORS AND RELATED PROTEINS**

**Supplementary Table 1A: Factors associated with plasma IGF proteins**

| Factors | | IGF1  Mean$\pm$SD  | IGF2  Mean$\pm$SD  | Log IGFBP1  Mean$\pm$SD  | IGFBP2  Median (IQR)^a^ | Log IGF2R  Mean$\pm$SD  |
| --- | --- | --- | --- | --- | --- | --- |
| Gender | Male (n=93)  Female (n=14) | 195.63 $\pm$ 55.68  171.61 $\pm$ 49.28  | 703.86 $\pm$ 170.31  699.45 $\pm$ 131.19  | 1.43 $\pm$ 0.75  1.45 $\pm$ 0.67  | 32.11 (15.72-51.76)  40.29 (6.54-47.68) | 4.45 $\pm$ 0.90  4.86 $\pm$ 0.61  |
| Race | Black (n=34)  Other (n=14)  White (n=59) | 190.23 $\pm$ 56.91  193.51 $\pm$ 54.26  193.53 $\pm$ 55.52  | 674.34 $\pm$ 182.87  686.95 $\pm$ 173.79  723.50 $\pm$ 152.30  | 1.35 $\pm$ 0.80  1.19 $\pm$ 0.55  1.54 $\pm$ 0.73  | 24.57 ( 3.90-45.44) *  15.08 ( 6.54-44.26)  40.84 (22.09-54.39) | 4.30 $\pm$ 0.94  4.87 $\pm$ 0.60  4.54 $\pm$ 0.88  |
| AIDS | No (N=39)  Yes (n = 68) | 205.58 $\pm$ 52.97 ^b^  184.83 $\pm$ 55.54  | 701.65 $\pm$ 141.58  704.25 $\pm$ 178.70  | 1.42 $\pm$ 0.74  1.43 $\pm$ 0.74  | 33.04 (14.20-47.70)  36.31 (14.44-52.28) | 4.41 $\pm$ 0.88  4.56 $\pm$ 0.88  |
| ART use | No (n=23)  Yes (n=84) | 204.57 $\pm$ 61.75  189.08 $\pm$ 53.18  | 661.56 $\pm$ 114.25 ^d^  715.21 $\pm$ 175.88  | 1.44 $\pm$ 0.71  1.43 $\pm$ 0.75  | 28.78 (3.98-55.62)  34.02 (15.72-47.72) | 4.64 $\pm$ 0.67  4.47 $\pm$ 0.93  |
| Plasma VL | Non-detectable (n=53)  Detectable (n=54) | 195.02 $\pm$ 56.49  190.20 $\pm$ 54.54  | 725.59 $\pm$ 169.60  681.79 $\pm$ 159.52  | 1.30 $\pm$ 0.70 ^e^  1.55 $\pm$ 0.76  | 28.29 (11.85-47.68)  37.06 (14.44-54.39) | 4.22 $\pm$ 0.94 ***  4.77 $\pm$ 0.73  |
| Current CD4 | < 200 (n=22)  >= 200 (n=85) | 187.06 $\pm$ 58.48  193.78 $\pm$ 55.01  | 670.57 $\pm$ 202.26  711.20 $\pm$ 155.51  | 1.77 $\pm$ 0.79 *  1.35 $\pm$ 0.70  | 37.06 (1.93-54.39)  32.11 (15.72-47.72) | 4.74 $\pm$ 0.94  4.43 $\pm$ 0.85  |
| Body mass index (BMI) | BMI <= 25 (n=44)  25 < BMI < 30 (n=45)  BMI >= 30 (n=17) | 203.31 $\pm$ 51.43 ^c^  187.68 $\pm$ 55.00  176.27 $\pm$ 64.53  | 694.74 $\pm$ 156.13  697.06 $\pm$ 164.63  741.17 $\pm$ 197.89  | 1.78 $\pm$ 0.74***  1.33 $\pm$ 0.56  0.88 $\pm$ 0.67  | 37.24 (16.37-55.66)  30.51 (15.72-47.72)  33.97 (6.54-45.44) | 4.66 $\pm$ 0.77  4.42 $\pm$ 0.92  4.34 $\pm$ 1.04  |
| Albumin | 3 or 4 (n=72)  5 (n=34) | 191.94 $\pm$ 54.87  195.79 $\pm$ 56.62  | 704.01 $\pm$ 153.09  715.51 $\pm$ 172.80  | 1.42 $\pm$ 0.71  1.43 $\pm$ 0.75  | 30.51 (11.86-54.48)  37.06 (20.30-47.72) | 4.53 $\pm$ 0.84  4.40 $\pm$ 0.96  |
| Alcohol dependence | No (n=82)  Yes (n=25) | 188.58 $\pm$ 55.64  205.60 $\pm$ 53.06  | 698.47 $\pm$ 171.54  719.25 $\pm$ 144.40  | 1.42 $\pm$ 0.77  1.45 $\pm$ 0.64  | 28.29 (9.25-47.68)  37.41 (22.09-54.39) | 4.50 $\pm$ 0.89  4.51 $\pm$ 0.85  |

^a^IQR: interquartile range.

* p<0.05, *** p<0.001. b: p=0.059, c: p=0.062, d: p=0.081. e: p=0.074

**Supplementary Table 1B: Correlation of plasma IGF proteins**

| *plasma factors*  *(n=107)* | *IGF1* | | *IGF2* | | *IGFBP1* | | *IGFBP2* | | *IGF2R* | |
| --- | --- | --- | --- | --- | --- | --- | --- | --- | --- | --- |
|  | *r* | *p value* | *r* | *p value* | *r* | *p value* | *r* | *p value* | *r* | *p value* |
| Age | -0.21 | 0.030 | 0.07 | 0.478 | 0.01 | 0.916 | 0.13 | 0.178 | -0.10 | 0.286 |
| IGF2R | -0.07 | 0.483 | -0.03 | 0.755 | 0.21 | 0.029 | 0.26 | 0.007 | NA | NA |
| IGF1 | NA | NA | 0.40 | <0.001 | -0.08 | 0.423 | -0.00 | 0.976 | -0.07 | 0.483 |
| IGF2 | 0.40 | <0.001 | NA | NA | -0.05 | 0.598 | 0.05 | 0.614 | -0.03 | 0.755 |
| IGFBP-1 | -0.08 | 0.423 | -0.14 | 0.155 | NA | NA | 0.30 | 0.002 | 0.21 | 0.029 |
| IGFBP-2 | -0.00 | 0.976 | 0.10 | 0.319 | 0.30 | 0.002 | NA | NA | 0.26 | 0.007 |
| Current CD4+ | -0.01 | 0.891 | 0.10 | 0.293 | -0.19 | 0.058 | -0.09 | 0.378 | -0.20 | 0.042 |
| IFNγ | -0.10 | 0.311 | 0.11 | 0.271 | 0.04 | 0.733 | -0.4 | 0.694 | 0.19 | 0.055 |
| IL-6 | -0.18 | 0.069 | -0.18 | 0.070 | 0.09 | 0.389 | 0.21 | 0.032 | 0.19 | 0.049 |
| IL-10 | -0.14 | 0.164 | -0.10 | 0.323 | 0.18 | 0.064 | 0.14 | 0.138 | 0.26 | 0.006 |
| IL-17 | -0.17 | 0.081 | -0.00 | 0.971 | 0.21 | 0.028 | 0.03 | 0.758 | 0.34 | <0.001 |
| IP-10 | 0.00 | 0.991 | -0.25 | 0.011 | 0.15 | 0.130 | 0.03 | 0.736 | -0.06 | 0.565 |
| MCP-1 | -0.04 | 0.667 | -0.04 | 0.689 | 0.14 | 0.156 | 0.18 | 0.063 | 0.23 | 0.016 |
| TNFá | -0.11 | 0.278 | -0.03 | 0.802 | 0.21 | 0.030 | 0.13 | 0.189 | 0.34 | <0.001 |
| Progranulin | -0.09 | 0.375 | 0.15 | 0.128 | 0.29 | 0.003 | 0.02 | 0.842 | 0.11 | 0.278 |
| AST | -0.18 | 0.069 | -0.03 | 0.734 | 0.01 | 0.979 | 0.06 | 0.535 | 0.02 | 0.869 |
| ALT | -0.10 | 0.325 | 0.04 | 0.689 | -0.17 | 0.082 | 0.05 | 0.638 | 0.05 | 0.637 |

r: Spearman correlation coefficient

NA: not applicable

**Supplementary Table 2A: Factors associated with CSF IGF proteins**

| Factors | | IGF1  Median(Q1-Q3) | Log IGF2  Mean$\pm$SD  | IGFBP1  Median (IQR)^a^ | Log IGFBP2  Mean$\pm$SD  | IGF2R ^0.5^  Mean$\pm$SD  |
| --- | --- | --- | --- | --- | --- | --- |
| Gender | Male (n=93)  Female (n=14) | 1.12 (0.92-1.50)  1.03 (0.80-1.29) | 3.71 $\pm$ 0.17  3.63 $\pm$ 0.17  | 0.48 (0.37-0.77)  0.39 (0.23-0.65) | 4.38 $\pm$ 0.37 *  4.18 $\pm$ 0.31  | 2.54 $\pm$ 0.85  2.69 $\pm$ 0.80  |
| Race | Black (n=34)  Other (n=14)  White (n=59) | 0.92 (0.73-1.29) *  1.09 (0.95-1.48)  1.22 (0.99-1.71) | 3.63 $\pm$ 0.15 **  3.65 $\pm$ 0.14  3.75 $\pm$ 0.17  | 0.46 (0.33-0.82)  0.41 (0.33-1.62)  0.48 (0.37-0.75) | 4.25 $\pm$ 0.42  4.36 $\pm$ 0.24  4.41 $\pm$ 0.35  | 2.27 $\pm$ 0.75 *  3.01 $\pm$ 0.76  2.62 $\pm$ 0.86 |
| AIDS | No (N=39)  Yes (n = 68) | 1.29 (0.88-1.67)  1.07 (0.92-1.44) | 3.68 $\pm$ 0.16  3.71 $\pm$ 0.18 | 0.44 (0.34-0.65) *  0.49 (0.36-1.17) | 4.33 $\pm$ 0.33  4.37 $\pm$ 0.39 | 2.39 $\pm$ 0.86  2.66 $\pm$ 0.82 |
| ART use | No (n=23)  Yes (n=84) | 1.29 (0.92-1.67)  1.10 (0.88-1.48) | 3.65 $\pm$ 0.16  3.71 $\pm$ 0.17 | 0.44 (0.37-0.72)  0.49 (0.35-0.80) | 4.32 $\pm$ 0.35  4.36 $\pm$ 0.37 | 2.28 $\pm$ 0.83 ^b^  2.64 $\pm$ 0.83 |
| CSF VL | Non-detectable (n=79)  Detectable (n=28) | 1.10 (0.88-1.48)  1.14 (0.92-1.50) | 3.71 $\pm$ 0.17  3.69 $\pm$ 0.18 | 0.44 (0.33-0.75)  0.51 (0.38-1.31) | 4.35 $\pm$ 0.37  4.37 $\pm$ 0.38 | 2.60 $\pm$ 0.85  2.45 $\pm$ 0.82 |
| Current CD4 | < 200 (n=22)  >= 200 (n=84) | 1.03 (0.88-1.41)  1.14 (0.92-1.56) | 3.70 $\pm$ 0.20  3.70 $\pm$ 0.16 | 0.46 (0.35-0.77)  0.48 (0.37-0.75) | 4.41 $\pm$ 0.50  4.34 $\pm$ 0.33 | 2.60 $\pm$ 0.75  2.54 $\pm$ 0.87 |
| Body mass index | BMI <= 25 (n=44)  25 < BMI < 30 (n=45)  BMI >= 30 (n=17) | 1.11 (0.92-1.44)  1.10 (0.80-1.56)  1.22 (0.95-1.48) | 3.69 $\pm$ 0.14  3.70 $\pm$ 0.20  3.69 $\pm$ 0.18 | 0.60(0.46-1.53)***  0.41 (0.33-0.66)  0.37 (0.23-0.40) | 4.38 $\pm$ 0.35  4.34 $\pm$ 0.38  4.33 $\pm$ 0.40 | 2.64 $\pm$ 0.67  2.40 $\pm$ 0.96  2.74 $\pm$ 0.87 |
| Albumin | 3 or 4 (n=71)  5 (n=34) | 1.11 (0.88-1.52)  1.11 (0.92-1.48) | 3.70 $\pm$ 0.17  3.70 $\pm$ 0.18 | 0.48 (0.37-1.50) ^c^  0.44 (0.30-0.62) | 4.40 $\pm$ 0.39 *  4.25 $\pm$ 0.31 | 2.41 $\pm$ 0.84 *  2.85 $\pm$ 0.76 |
| Alcohol dependence | No (n=82)  Yes (n=25) | 1.07 (0.88-1.48) ^d^  1.29 (1.03-1.82) | 3.71 $\pm$ 0.17  3.66 $\pm$ 0.19 | 0.43 (0.34-0.72) ^e^  0.64 (0.40-1.62) | 4.35 $\pm$ 0.35  4.38 $\pm$ 0.41 | 2.65 $\pm$ 0.84 *  2.27 $\pm$ 0.79 |

^a^IQR: interquartile range

* p<0.05, ** p<0.01, *** p<0.001, b: p=0.070, c: p=0.085, d: p=0.063, e: p=0.057

**Supplementary Table 2B: Correlation of CSF IGF proteins**

| *CSF*  *factors*  *(n=107)* | *IGF1* | | *IGF2* | | *IGFBP1* | | *IGFBP2* | | *IGF2R* | |
| --- | --- | --- | --- | --- | --- | --- | --- | --- | --- | --- |
|  | *r* | *p value* | *r* | *p value* | *r* | *p value* | *r* | *p value* | *r* | *p value* |
| Age | 0.16 | 0.096 | 0.20 | 0.036 | 0.09 | 0.364 | -0.02 | 0.841 | 0.23 | 0.017 |
| IGF2R | 0.03 | 0.748 | 0.19 | 0.046 | -0.08 | 0.412 | 0.04 | 0.669 | NA | NA |
| IGF1 | NA | NA | 0.20 | 0.041 | -0.04 | 0.694 | 0.10 | 0.300 | 0.03 | 0.748 |
| IGF2 | 0.20 | 0.041 | NA | NA | 0.17 | 0.086 | 0.34 | <0.001 | 0.19 | 0.046 |
| IGFBP-1 | -0.04 | 0.694 | 0.17 | 0.086 | NA | NA | 0.28 | 0.004 | -0.08 | 0.412 |
| IGFBP-2 | 0.10 | 0.300 | 0.34 | <0.001 | 0.28 | 0.004 | NA | NA | 0.04 | 0.668 |
| Current CD4+ | 0.06 | 0.532 | -0.07 | 0.489 | -0.04 | 0.656 | 0.02 | 0.814 | -0.15 | 0.117 |
| IL-6 | 0.14 | 0.138 | 0.05 | 0.591 | 0.18 | 0.061 | 0.25 | 0.011 | -0.06 | 0.542 |
| IL-10 | 0.21 | 0.034 | 0.20 | 0.036 | 0.21 | 0.031 | 0.24 | 0.011 | -0.04 | 0.731 |
| IP-10 | 0.14 | 0.143 | 0.25 | 0.009 | 0.20 | 0.036 | 0.22 | 0.022 | 0.02 | 0.802 |
| MCP-1 | -0.02 | 0.864 | -0.01 | 0.955 | 0.002 | 0.868 | 0.11 | 0.260 | -0.04 | 0.705 |
| TNFá | 0.09 | 0.336 | 0.24 | 0.012 | 0.40 | <0.001 | 0.32 | <0.001 | 0.06 | 0.533 |
| progranulin | 0.26 | 0.008 | 0.15 | 0.123 | 0.20 | 0.045 | 0.14 | 0.157 | 0.16 | 0.103 |
| AST-SGOT | -0.07 | 0.450 | -0.08 | 0.439 | -0.04 | 0.703 | -0.17 | 0.075 | 0.15 | 0.128 |
| ALT-SGPT | -0.01 | 0.913 | 0.04 | 0.654 | -0.16 | 0.105 | -0.14 | 0.164 | 0.22 | 0.025 |

r :Spearman correlation coefficient

NA: not applicable

**Supplementary Table 3: IGFs and related protein levels stratified by ART regimen**

|  | | NNRTI based (n=36) | PI based (n=40) | p value |
| --- | --- | --- | --- | --- |
| plasma | IGF1 (mean ± SD, ng/ml) | 174.1 ± 59.8 | 202.8 ± 47.3 | 0.039 |
|  | IGF2 (mean ± SD, ng/ml) | 695.4 ± 186.2 | 729.4 ± 174.6 | 0.389 |
|  | ^a^log IGFBP1 (mean ± SD, ng/ml) | 1.41 ± 0.82 | 1.50 ± 0.73 | 0.624 |
|  | IGFBP2 – median (IQR) ^c^ | 29.98 (18.60-45.44) | 44.26 (9.25-65.57) | 0.154 |
|  | ^a^log IGF2R (mean ± SD, ng/ml) | 4.47 ± 0.99 | 4.47 ± 0.95 | 0.989 |
| CSF | IGF1 – median (IQR)^c^ | 1.05 (0.83-1.49) | 1.15 (0.92-1.48) | 0.318 |
|  | ^a^log IGF2 (Mean ± SD, ng/ml) | 3.70 ± 0.15 | 3.74 ± 0.18 | 0.292 |
|  | IGFBP1 – median (IQR) ^c^ | 0.54 (0.35-1.63) | 0.43 (0.33-0.71) | 0.474 |
|  | ^a^log IGFBP2 (mean ± SD, ng/ml) | 4.32 ± 0.30 | 4.41 ± 0.44 | 0.289 |
|  | ^b^IGF2R^0.5^ (mean ± SD, ng/ml) | 2.62 ± 0.85 | 2.70 ± 0.80 | 0.683 |

^a^Plasma IGFBP1, plasma IGF2R, CSF IGF2 and CSF IGFBP2 were transformed with natural logarithms

^b^CSF IGF2R was square root transformed

IQR^c^ : interquartile range,
